# The suppressor of copper sensitivity protein C from *Caulobacter crescentus* is a trimeric disulfide isomerase that binds copper(I) with subpicomolar affinity

**DOI:** 10.1107/S2059798322000729

**Published:** 2022-02-21

**Authors:** Guillaume A. Petit, Yaoqin Hong, Karrera Y. Djoko, Andrew E. Whitten, Emily J. Furlong, Airlie J. McCoy, Jacqueline M. Gulbis, Makrina Totsika, Jennifer L. Martin, Maria A. Halili

**Affiliations:** a Griffith Institute for Drug Discovery, Griffith University, Don Young Road, Nathan, QLD 4111, Australia; b Centre for Immunology and Infection Control and School of Biomedical Sciences, Queensland University of Technology, Herston, QLD 4006, Australia; c Department of Biosciences, Durham University, Durham DH1 3LE, United Kingdom; d Australian Nuclear Science and Technology Organization (ANSTO), New Illawarra Road, Lucas Heights, NSW 2234, Australia; e Institute for Molecular Bioscience, The University of Queensland, St Lucia, Queensland 4072, Australia; f Department of Haematology, Cambridge Institute for Medical Research, University of Cambridge, Cambridge CB2 0XY, United Kingdom; g Structural Biology Division, The Walter and Eliza Hall Institute of Medical Research, Parkville, VIC 3052, Australia; h Department of Medical Biology, The University of Melbourne, Parkville, VIC 3052, Australia; i Vice-Chancellor’s Unit, University of Wollongong, Building 36, Wollongong, NSW 2522, Australia

**Keywords:** suppressor of copper sensitivity protein C, X-ray crystallography, small-angle X-ray scattering, disulfide bond-forming proteins, protein trimers, copper-binding proteins

## Abstract

The introduction of disulfide bonds into periplasmic proteins is a critical process in many Gram-negative bacteria. The formation and regulation of protein disulfide bonds have been linked to the production of virulence factors. Understanding the different pathways involved in this process is important in the development of strategies to disarm pathogenic bacteria. The well characterized disulfide bond-forming (DSB) proteins play a key role by introducing or isomerizing disulfide bonds between cysteines in substrate proteins. Curiously, the suppressor of copper sensitivity C proteins (ScsCs), which are part of the bacterial copper-resistance response, share structural and functional similarities with DSB oxidase and isomerase proteins, including the presence of a catalytic thioredoxin domain. However, the oxidoreductase activity of ScsC varies with its oligomerization state, which depends on a poorly conserved N-terminal domain. Here, the structure and function of *Caulobacter crescentus* ScsC (CcScsC) have been characterized. It is shown that CcScsC binds copper in the copper(I) form with subpicomolar affinity and that its isomerase activity is comparable to that of *Escherichia coli* DsbC, the prototypical dimeric bacterial isomerase. It is also reported that CcScsC functionally complements trimeric *Proteus mirabilis* ScsC (PmScsC) *in vivo*, enabling the swarming of *P. mirabilis* in the presence of copper. Using mass photometry and small-angle X-ray scattering (SAXS) the protein is demonstrated to be trimeric in solution, like PmScsC, and not dimeric like EcDsbC. The crystal structure of CcScsC was also determined at a resolution of 2.6 Å, confirming the trimeric state and indicating that the trimerization results from interactions between the N-terminal α-helical domains of three CcScsC protomers. The SAXS data analysis suggested that the protomers are dynamic, like those of PmScsC, and are able to sample different conformations in solution.

## Abstract 2

The characterization of the suppressor of copper sensitivity protein C from *C. crescentus* is reported.

## 1. Introduction

Disulfide bonds are essential for many exported proteins to achieve their functional native state and to increase their stability. As a result, bacteria have developed systems to catalyse and regulate the formation of protein disulfide bonds within their periplasm and outer membranes. To date, the best-characterized of these systems is the disulfide bond-forming (DSB) protein system, which has been thoroughly studied in *Escherichia coli* K-12 (Landeta *et al.*, 2018 ▸; Hatahet *et al.*, 2014 ▸; Ito & Inaba, 2008 ▸; Gleiter & Bardwell, 2008 ▸). It consists of the DsbA–DsbB redox pair, which is responsible for the formation of disulfide bonds, and the DsbC–DsbD protein pair, which isomerizes or reduces incorrectly formed disulfides (Veenendaal *et al.*, 2004 ▸; Messens & Collet, 2006 ▸; Bader *et al.*, 1999 ▸). The functions of both the monomeric DsbA and the dimeric DsbC require a thioredoxin-fold catalytic domain (Holmgren *et al.*, 1975 ▸; Martin, 1995 ▸). The oxidation, reduction or isomerization reactions catalysed by the DsbA and DsbC proteins are conducted by the transfer of disulfide bonds from or to the C*XX*C active-site catalytic motif of the thioredoxin fold (Zapun *et al.*, 1995 ▸; Bardwell, 1994 ▸; Wunderlich & Glockshuber, 1993 ▸), where C represents the two conserved cysteine residues and *X* represents any other amino acid.

Recently, a novel family of proteins have been shown to regulate disulfide-bond formation in the periplasm of bacteria: the suppressor of copper sensitivity proteins (SCSs; Furlong *et al.*, 2018 ▸; Subedi *et al.*, 2019 ▸; Shepherd *et al.*, 2013 ▸). First reported in *Salmonella enterica* serovar Typhimurium (*S.* Typhimurium), the SCS protein family consists of four members (ScsA–ScsD) encoded in one locus (Gupta *et al.*, 1997 ▸). Little is known about ScsA and ScsD, while ScsB and ScsC have now been characterized in several bacterial species (Subedi *et al.*, 2019 ▸; Furlong *et al.*, 2017 ▸, 2018 ▸, 2019 ▸; Shepherd *et al.*, 2013 ▸; Cho *et al.*, 2012 ▸; Gupta *et al.*, 1997 ▸). ScsB is a relatively large protein comprising three domains: an N-terminal α-domain and a C-terminal γ-domain, which are both located in the periplasm and are separated by a transmembrane β-domain. In *Proteus mirabilis*, *S.* Typhimurium and *Caulobacter crescentus*, ScsB is necessary to maintain the active-site cysteines of ScsC in their reduced dithiol active state (Subedi *et al.*, 2019 ▸; Furlong *et al.*, 2018 ▸; Cho *et al.*, 2012 ▸).

**Figure 1:**
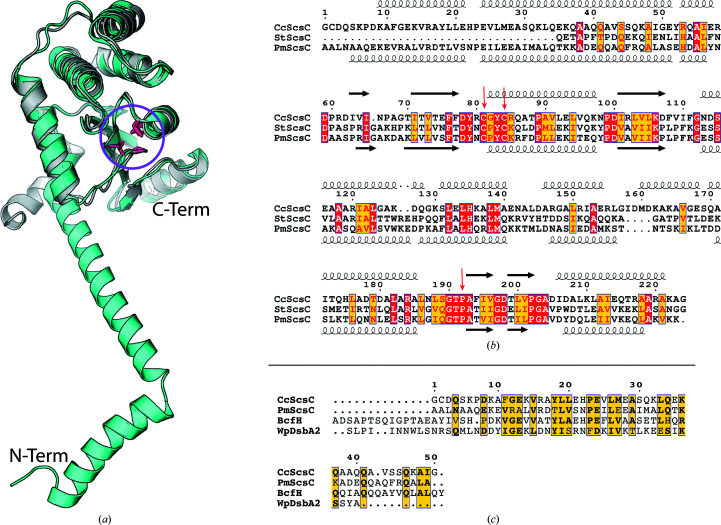
ScsC sequence comparison. (*a*) Cartoon representation of the structural superposition of the catalytic domain of PmScsC (cyan, PDB entry 5id4; Furlong *et al.*, 2017 ▸) with StScsC (grey, PDB entry 4gxz; Shepherd *et al.*, 2013 ▸). The globular catalytic domains of these two proteins align well (r.m.s.d. of 1.7 Å for 174 C^α^ atoms aligned using *TM-align*; Zhang & Skolnick, 2005 ▸). However, PmScsC has a long N-terminal domain involved in its trimerization that is absent in StScsC. The N-terminus of the PmScsC model is labelled N-Term and the C-terminus is labelled C-Term. The catalytic cysteines and an adjacent threonine–*cis*-proline sequence of the thioredoxin domain are shown as pink sticks (highlighted with a pink circle). (*b*) Sequence alignment of mature (no signal sequence) CcScsC (UniProt ID Q9A747), StScsC (UniProt ID H9L4C1) and PmScsC (UniProt ID B4EV21). The catalytic domains of the three sequences share high similarity (PmScsC shares 25% sequence identity with CcScsC and 53% sequence identity with StScsC). CcScsC and PmScsC both have an extra N-terminal domain which is absent in StScsC. Secondary-structure annotation based on the structure of CcScsC presented in this work is shown above the sequence alignment. Secondary-structure annotation based on the structure of PmScsC (PDB entry 5id4) is shown below the alignment: coils for α-helices and arrows for β-strands. Note that the first two residues as well as a seven-residue C-terminal TEV protease cleavage scar differ between the CcScsC UniProt sequence and the protein sequence used for crystallization. Similar residues are highlighted in yellow, identical residues are highlighted in red and catalytic cysteines and *cis*-prolines are highlighted with red arrows. (*c*) The N-terminal region (50 residues after the signal peptide) of CcScsC was compared with those of other known trimeric thioredoxin-fold proteins: PmScsC, StBcfH (UniProt ID A0A0H3N7J9, 65 residues beyond the signal sequence selected) and WpDsbA2 (UniProt ID Q73FL6). This alignment reveals a similarity between the N-terminal sequences of the different proteins (17.5% identity between CcScsC and WpDsbA2, 30% identity between CcScsC and PmScsC, 36% identity between CcScsC and StBcfH). Two large gaps are the consequence of the additional residues of StBcfH.

*S.* Typhimurium ScsC (StScsC) is a monomeric, soluble protein localized to the periplasm that restores copper tolerance to copper-sensitive *Escherichia coli* cells (Gupta *et al.*, 1997 ▸). StScsC binds copper(I) with subpicomolar affinity and interacts with the copper-binding metallochaperone CueP (Subedi *et al.*, 2019 ▸; Shepherd *et al.*, 2013 ▸; Osman *et al.*, 2013 ▸). Despite strong structural resemblance to DsbA enzymes, it has neither oxidase nor isomerase activity (Subedi *et al.*, 2019 ▸; Shepherd *et al.*, 2013 ▸). On the other hand, *C. crescentus* ScsC (CcScsC) and *P. mirabilis* ScsC (PmScsC) have both been reported to be protein disulfide isomerases (Cho *et al.*, 2012 ▸; Furlong *et al.*, 2017 ▸). The three ScsC proteins that have been characterized to date (StScsC, CcScSC and PmScsC) all have a catalytic thioredoxin domain including catalytic cysteines in a C*XX*C active-site motif. However, the PmScsC and CcScsC sequences both have a long N-terminal region which is absent in StScsC (Figs. 1 ▸ *a* and 1 ▸ *b*). Overall, the ScsB–ScsC redox couple shares structural and functional similarities with DsbD and DsbC (Cho & Collet, 2013 ▸).

Crystal structures of PmScsC revealed that the N-terminal region is α-helical and supports trimerization (Fig. 1 ▸ *a*; Furlong *et al.*, 2017 ▸, 2019 ▸). By comparison, and based on size-exclusion chromatography (SEC) analysis, CcScsC has been reported to be dimeric, eluting in a peak corresponding to 47 ± 10 kDa (Cho *et al.*, 2012 ▸; the monomeric protein is approximately 25 kDa). However, the sequence of CcScsC is more closely related overall to that of the trimeric protein PmScsC (25% sequence identity and 49% sequence similarity) than to that of the dimeric EcDsbC (17% sequence identity and 30% sequence similarity). Moreover, the putative N-terminal oligomerization domains (50 residues after the signal sequence of both PmScsC and CcScsC) share more than 30% sequence identity. Comparison of the N-terminal sequence of CcScsC with those of other bacterial oxidoreductases that have been reported to be trimeric [*S.* Typhimurium BcfH (StBcfH; Subedi *et al.*, 2021 ▸) and *Wolbachia pipientis* α-DsbA2 (WpDsbA2; Walden *et al.*, 2019 ▸)] further highlights similarities between the trimeric proteins and CcScsC (Fig. 1 ▸ *c*). Alignment of the N-terminal regions of the mature (no signal peptide) sequences of PmScsC, StBcfH, WpDsbA2 and CcScsC reveals that while their sequence identity is low, there are short segments of hydrophobic and charged residues that are similarly positioned across all four proteins (Fig. 1 ▸ *c*). Consequently, we hypothesized that CcScsC is trimeric, not dimeric.

The *C. crescentus* genome encodes two putative DsbA proteins and one putative DsbB protein (a putative redox partner of DsbA), but no equivalent of DsbC. Accordingly, CcScsC has been proposed to be the *in vivo C. crescentus* disulfide isomerase enzyme (Cho *et al.*, 2012 ▸). Here, we report the detailed functional and structural characterization of CcScsC. We show that CcScsC is a disulfide isomerase with an equivalent activity to that of EcDsbC (as is the C2S variant CcScsC used for crystal structure determination), complements PmScsC in *P. mirabilis* swarming assays and binds copper with subpicomolar affinity. We also show using mass photometry and small-angle X-ray scattering (SAXS) analysis that CcScsC forms trimers in solution. The crystal structure of CcScsC, solved to a resolution of 2.63 Å, confirms that the protein is trimeric and that the trimer is formed through the interaction of the N-terminal regions of three protomers. The SAXS data suggest that, like PmScsC, CcScsC is highly dynamic in solution.

## 2. Materials and methods

### 2.1. Sequence analysis

The sequences of CcScsC (UniProt ID Q9A747), EcDsbC (UniProt ID P0AEG6), PmScsC (UniProt ID B4EV21), StScsC (UniProt ID H9L4C1), StBcfH (UniProt ID A0A0H3N7J9) and WpDsbA2 (UniProt ID Q73FL6), not including their respective signal peptides, were aligned with *EMBOSS Needle* (Madeira *et al.*, 2019 ▸) for pairwise alignments or *Clustal Omega* (Madeira *et al.*, 2019 ▸) for multiple sequence alignment, using the default parameters. Figures were prepared using *ESPript* 3.0 (Robert & Gouet, 2014 ▸).

### 2.2. CcScsC constructs

The gene for *C. crescentus* ScsC (UniProt ID Q9A747) was codon-optimized for *E. coli* expression and was ordered from GenScript, Piscataway, USA.

Two constructs were designed: one coding for the wild-type CcScsC protein (CcScsC wt) and one for a variant in which the cysteine at position 2 was changed to a serine (referred to as the C2S mutant) to facilitate characterization of only the catalytic cysteines. Primers for these constructs were ordered from IDT Integrated DNA Technology (Science Park II, Republic of Singapore). These primers were designed to remove the signal sequence from the gene and introduce NdeI (5′-GGA ATT CCA TAT GTG CGA CCA AAG CAA GCC GG-3′) and XhoI (5′-CCG CTC GAG GCC CGC TTT CGC ACG CGC-3′) restriction sites. Finally, one primer allowed the C2S mutation (5′-GGA ATT CCA TAT GAG CGA CCA AAG CAA GCC GGA C-3′), producing a variant referred to as CcScsC C2S.

The CcScsC sequences (C2S and wt) were inserted into a pET-24a vector in front of a His_10_ tag attached to a C-terminal linker containing a Tobacco etch virus protease (TEV protease) cleavage site and transformed in *E. coli* BL21(DE3) pLysS cells (Invitrogen). The final protein sequences therefore differed from the UniProt sequence by starting with a methionine instead of a glycine and by having seven residues left over from the TEV protease cleavage scar at the C-terminus (^225^LEENLYF^231^) as well as the C2S mutation in CcScsC C2S. The cultures were grown for 20 h at 30°C in ZYP-5052 autoinduction medium (Studier, 2005 ▸) containing kanamycin at 50 µg ml^−1^ and chloramphenicol at 34 µg ml^−1^. The cells were harvested by centrifugation for 15 min at 6000*g*; the resulting cell pellets (around 15 g per litre of culture) were frozen and stored at −80°C until purification.

The purification protocol was the same for the two variants of the protein. The cell pellet from 1 l cell culture was resuspended in 300 ml Tris buffer (25 m*M* Tris pH 7.5, 150 m*M* NaCl) mixed with 150 µl DNase I solution (6.7 mg ml^−1^, Roche) and 200 µl EDTA-free protease-inhibitor cocktail Set III (Merck). The cells were passed twice through a cell disruptor (Constant Systems) at 150 kPa. The lysate was centrifuged for 30 min at 40 000*g* at 4°C and the cell debris (pellet) was discarded. Imidazole was added to the supernatant containing the protein to a final concentration of 20 m*M* and this solution was mixed with 10 ml nickel–nitrilo­triacetic acid (Ni–NTA) agarose (Qiagen) equilibrated in HEPES–imidazole buffer (25 m*M* HEPES, 20 m*M* imidazole, 150 m*M* NaCl). The protein/Ni–NTA mixture was loaded onto two 25 ml gravity-flow columns. The flowthrough was collected and passed through the resin a second time. The resin was washed with 30 ml HEPES–imidazole buffer and the protein was eluted with 30 ml elution buffer (25 m*M* HEPES pH 7.5, 150 m*M* NaCl, 250 m*M* imidazole) and concentrated to 10 ml using Amicon ultracentrifugal filters (Millipore). To remove the imidazole, the protein solution was injected onto a desalting column (GE 25 Superfine) 5 ml at a time using a Bio-Rad NGC FPLC system. After desalting, the protein solution was mixed with 5 mg TEV protease and incubated for 30 min at room temperature (RT) and then overnight at 4°C to cleave the His_10_ tag.

The overnight incubation with TEV protease led to a large amount of visible precipitate, most of which was the His_10_ tag cleaved from the protein according to SDS–PAGE analysis. The protein solution was centrifuged for 10 min at 3500*g* to remove the precipitate. The supernatant was once again loaded onto 5 ml pre-equilibrated Ni–NTA resin and the protein–resin mixture was loaded onto gravity-flow columns. TEV protease-cleaved protein lacking the His tag was eluted from the column with 15 ml SEC buffer (25 m*M* HEPES pH 7.5, 150 m*M* NaCl). At this stage the protein was reduced. Reduction was performed by adding dithiothreitol (DTT) to the protein solution to a final concentration of 5 m*M* and incubating at RT for 1 h. Finally, DTT was removed from the protein solution by SEC using a Superdex S200 16/600 gel-filtration column equilibrated in SEC buffer on a Bio-Rad NGC FPLC. The purity of the protein was assessed by SDS–PAGE analysis and the protein concentration was determined using a NanoDrop ND-1000 (Thermo Fisher). The oxidation state of the protein was assessed using the Ellman reaction (Ellman, 1959 ▸; Riddles *et al.*, 1983 ▸). The protein was concentrated as required (to 130 mg ml^−1^ for crystallization) using an Amicon 10 kDa molecular-weight cutoff centrifugal filter, flash-frozen in liquid nitrogen and stored at −80°C.

### 2.3. RNase-isomerization assay

This assay was performed to measure the ability of CcScsC wt and CcScsC C2S to isomerize misfolded RNase III from bovine pancreas. The protocol was run as described previously (Furlong *et al.*, 2017 ▸; Christensen *et al.*, 2016 ▸). RNase contains four disulfide bonds, which are all required for activity. Breaking and scrambling the disulfide bonds into random configurations results in inactive RNase. Bovine RNase type III (85% pure; Sigma–Aldrich) was reduced and denatured by mixing it with 6 *M* guanidinium hydrochloride (GdmCl) and 150 m*M* DTT in 50 m*M* Tris solution pH 8.0 and incubating for 28 h at RT. The DTT and GdmCl were removed by running the protein through a desalting column (GE 25 Superfine) on a Bio-Rad NGC FPLC system in 100 m*M* acetic acid/NaOH pH 4.0. An Ellman reagent test was used to verify that the protein was reduced. The protein was then left to air-oxidize in the dark for 5 d in 6 *M* GdmCl, 50 m*M* Tris pH 8.5. The GdmCl was removed by desalting into 100 m*M* sodium acetate pH 4.0 and the protein was concentrated to 10 mg ml^−1^ using 3 kDa molecular-weight cutoff Amicon Ultra centrifugal filters (Millipore). At this stage the RNase was shown by the Ellman test to be fully oxidized, with disulfide bonds formed randomly between its eight cysteines (making it mostly inactive).

**Figure 2:**
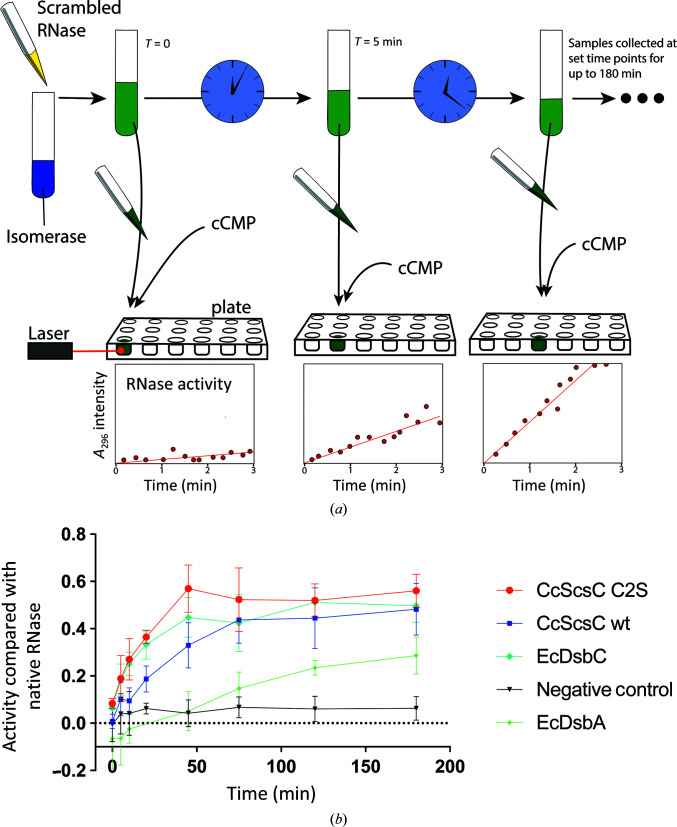
Scrambled RNase (ScRNase) activity assay. (*a*) Schematic of the assay. ScRNase (yellow solution) containing eight cysteines forming four randomly formed disulfide bonds is mixed with dithiol oxidase or disulfide isomerase (blue solution). Over time, the isomerase (such as EcDsbC) will correct the scrambled disulfide bonds in RNase to form native (active) RNase. At specific time points, samples of the RNase/enzyme solution (green solution) are taken and mixed with cCMP in a microplate (samples collected at 0, 5, 10, 20, 45, 75, 90, 120 and 180 min). The RNase activity is measured by monitoring the absorbance at 296 nm over a 3 min time period, where the reaction rate is the fastest and data points are in the linear range. Active RNase hydrolyses cCMP, increasing the absorbance (at 296 nm) of the solution. The bottom plots are illustrative schematics of the increasing activity of RNase in hydrolysing cCMP over the duration of the experiment. (*b*) Absorbance measurements are presented as a ratio of the activity of each enzyme tested relative to the activity of native RNase. CcScsC wt and its variant CcScsC C2S show isomerase activities comparable to that of the positive control EcDsbC. Each measurement corresponds to the mean activity value (*n* = 5, except for EcDsbA, where *n* = 2; error bars correspond to the standard deviation of the mean). EcDsbA was used as an oxidase enzyme control as it is expected to have moderate activity in this assay. The negative control contained ScRNase without any enzyme.

The RNAse-isomerization assay was performed as follows: samples of 10 µ*M* isomerase or oxidase (reduced CcScsC wt, CcScsC C2S and EcDsbC or oxidized EcDsbA as controls) were prepared in 1 m*M* EDTA, 100 m*M* sodium phosphate pH 7.0, 8.2 µ*M* DTT. The assay was started by adding 40 µ*M* scrambled RNase to these samples (Fig. 2 ▸ *a*). Scrambled RNase is isomerized to its native conformation by functional isomerases and becomes an active enzyme. At given time points (0, 5, 10, 20, 45, 75, 90, 120 and 180 min) 50 µl of the isomerase/oxidase + RNase solution was collected and mixed with 150 µl 4 m*M* cytidine cyclic 2′,3′-monophosphate (cCMP). The cleavage of cCMP by active RNase was determined by measuring the absorbance of the mixture at 296 nm. Each reaction was monitored over 3 min with a Synergy H1 plate reader (Biotek). An increasing absorbance at 296 nm (*A*_296_) over the 3 min signified that cCMP was being cleaved by active (and hence disulfide-isomerized) RNase. The results from the various samples were compared with controls (a positive control containing native RNase and no isomerase/oxidase and a negative control containing scrambled RNase and no isomerase). Activity was determined by comparing the *A*_296_ reaction rate (the slope of the curve over 3 min intervals) of each sample compared with native RNase and plotting the results against time incubated with the isomerases/oxidase (0, 5, 10, 20, 45, 75, 90, 120 and 180 min). The results presented are the mean and standard deviation over five replicates of each measurement for each sample, except for EcDsbA where only two replicates were collected.

### 2.4. Copper(I) binding by CcScsC

The copper(I) binding stoichiometry was determined by incubating 150 µ*M* CcScsC wt protein with two molar equivalents of copper(I) for 10 min in the presence of 2 m*M* sodium ascorbate (a reducing agent) in 50 m*M* 3-(*N*-morpholino)propanesulfonic acid (MOPS), 100 m*M* NaCl pH 7.4. Excess metal was removed with a PD-10 desalting column (Cytiva) using the same buffer as the eluent. The amount of protein present in each eluted fraction was determined by an Ellman test in the presence of 1 m*M* EDTA to chelate the copper ions, while the amount of copper(I) was determined colorimetrically using 1 m*M* bathocuproine disulfonic acid (BCS; the extinction coefficient ɛ_483_ of the [Cu^I^BCS_2_]^3−^ complex is 13 000 *M* ^−1^ cm^−1^). Both measurements were performed under denaturing conditions (6 *M* guanidinium–HCl).

The copper-binding affinity of CcScsC wt was determined following the methods described in Xiao *et al.* (2011 ▸) and Subedi *et al.* (2019 ▸). Briefly, CcScsC wt was competed with 2,2′-bicinchoninic acid (BCA) as described by the equilibrium reaction

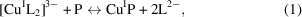

where P is the protein CcScsC wt, L is the BCA ligand and [Cu^I^L_2_]^3−^ corresponds to the [Cu^I^BCA_2_]^3−^ complex, which is measured colorimetrically (ɛ_562_ = 7900 *M* ^−1^ cm^−1^). In the experiment, the CcScsC wt protein (0–36.7 µ*M* final concentration) was titrated into a solution of 75 or 150 µ*M* [Cu^I^BCA_2_]^3−^ in 2 m*M* sodium ascorbate, 50 m*M* MOPS, 100 m*M* NaCl pH 7.4. Absorbance values at 562 nm were recorded and the data were fitted in *DynaFit* (BioKin) using a script that describes the equilibrium above. The *K*_d_ value for CcScsC wt was calculated using a log(β_2_) value of 17.3 for the [Cu^I^BCA_2_]^3−^ complex. The results were averaged over five replicates of the measurements.

### 2.5. Plasmid construction and strains for swarming assays

The *C. crescentus* *scsC* gene was amplified from pSC105 (pDSW204-Ec*dsbA*-ss-Δss-Cc*scsC*-His_6_; Cho *et al.*, 2012 ▸) using BamHI-Ec*dsbA*-F (5′-ACT GGG ATC CAT GAA AAA GAT TTG GCT GGC-3′) and HindIII-Cc*scsC*-R (5′-CGA TAA GCT TTC ACC CCG CTT TGG CCC GCG-3′) and then subcloned into pSU2718. The *P. mirabilis scsC* gene was previously cloned into pSU2718 (Furlong *et al.*, 2017 ▸). The two ScsC-expressing constructs and the empty vector pSU2718 were transformed into the previously constructed *P. mirabilis* PM54Δ*scsC* mutant (Furlong *et al.*, 2017 ▸) to give the strains PM54Δ*scsC*(pSU2718), PM54Δ*scsC*(pPm*scsC*) and PM54Δ*scsC*(pCc*scsC*).

### 2.6. Swarming motility on LB agar in the presence of copper(II)

Swarming-motility assays in the presence of copper were performed as described previously (Furlong *et al.*, 2017 ▸). Briefly, overnight cultures of *P. mirabilis* strains grown in the presence of 100 µ*M* isopropyl β-d-1-thiogalactopyranoside (IPTG) and 17 µg ml^−1^ chloramphenicol in LB Lennox were diluted to an OD_600_ of 0.1, and 10 µl was inoculated onto the centre of LB Lennox agar plates containing 1.6 m*M* CuSO_4_, 100 µ*M* IPTG and 17 µg ml^−1^ chloramphenicol. The plates were incubated at 37°C for 24 h and images were captured for analysis by *ImageJ*. The total swarmed surface area was measured in mm^2^ for each strain, data were normalized over the mean of the PM54Δ*scsC*(pSU2718) vector control (VC) and expressed as the percentage increase over the VC. Each strain was assessed in seven or eight biological replicates. The composition of LB Lennox (or LB Lennox agar) consisted of the following ingredients: 15 g bacteriological agar (BD Bacto, catalogue No. 214010, Lot 7009856), 5 g sodium chloride (Ajax Finechem, catalogue No. AJA465-5KG, Batch 1709250682), 5 g yeast extract (Amresco, catalogue No. J850-500G, Lot 1167C269) and 10 g tryptone (Gibco Bacto, catalogue No. 214010, Lot 0309043) per litre.

### 2.7. Swarming motility on LB agar without added NaCl

Swarming-motility assays under NaCl-free conditions were performed as described previously (Subedi *et al.*, 2021 ▸). Briefly, overnight cultures of *P. mirabilis* strains grown in the presence of 100 µ*M* IPTG and 17 µg ml^−1^ chloramphenicol in NaCl-free LB were streaked onto NaCl-free LB agar containing 100 µ*M* IPTG and 17 µg ml^−1^ chloramphenicol. The plates were incubated at 37°C for 24 h and images were captured on a ChemiDoc. Note that the inverse image display on the ChemiDoc was used for the image shown. Three independent experiments were performed. The composition of NaCl-free LB (or LB agar) consisted of the following ingredients: 15 g bacteriological agar (Oxoid, agar No. 1, catalogue No. LP0011, Lot 1451620-02), 5 g yeast extract (Oxoid, catalogue No. LP0021, Lot 1454768-02) and 10 g tryptone (Oxoid, catalogue No. LP0042, Lot 3110339) per litre.

### 2.8. Mass photometry

Mass-photometry analysis was performed at the Centre for Microscopy and Microanalysis at the University of Queensland on a Refeyn One mass photometer (Refeyn, Australia).

The instrument was blanked with buffer (25 m*M* HEPES pH 7.5, 150 m*M* NaCl). The measurements were calibrated using the NativeMark unstained protein standard (Thermo Fisher) diluted to 150 n*M* concentration. CcScsC samples were tested by diluting the protein to ∼150 n*M* and adding 2 µl of this protein solution at a time to 10 µl buffer reservoir until enough (>800) events (protein binding to the glass slide) were observed. The successive addition of small volumes of protein solution was necessary as each protein molecule bound to the glass slide differently. Data sets were recorded by monitoring the samples over 1 min, collecting 100 frames per second (6000 frames in total) with the *AcquireMP* software and setting the contrast to ±0.05. Analysis was performed automatically with *DiscoverMP*. Results are displayed as the mean ± standard deviation of the mean for the mass distribution of the main peak.

### 2.9. Small-angle X-ray scattering (SAXS)

SAXS data for CcScsC wt (residues 3–224) were collected on the SAXS/WAXS beamline at the Australian Synchrotron using an inline SEC-SAXS sheath-flow setup (Table 1 ▸; Kirby *et al.*, 2013 ▸, 2016 ▸). Data reduction was carried out using *scatterBrain* (software for acquiring, processing and viewing SAXS/WAXS data at the Australian Synchrotron) and corrected for solvent scattering and sample transmission. For the SEC-SAXS data, 50 × 1 s frames measured prior to the elution of the protein were averaged and taken as the solvent scattering. The sample scattering was taken as the average of 28 × 1 frames with similar *R*_g_ values that were measured as the protein eluted. For the solvent and sample scattering data, *CorMap* (Franke *et al.*, 2015 ▸) was used to look for systematic changes over the averaged ranges. The statistical pairwise comparison revealed no evidence of any changes in the scattering pattern over the averaged ranges. Contrast and partial specific volumes were determined from the protein sequences (Whitten *et al.*, 2008 ▸), while the molecular mass was estimated from the Porod volume (Fischer *et al.*, 2010 ▸). Data processing and Guinier analysis were performed using *PRIMUS* (Konarev *et al.*, 2003 ▸). The pair-distance distribution function [*p*(*r*)] was generated from the experimental data using *GNOM* (Svergun, 1999 ▸), from which *I*(0), *R*_g_ and *D*_max_ were determined. *CORAL* was used to generate 16 rigid-body models assuming a trimeric structure with *C*_3_ symmetry (Manalastas-Cantos *et al.*, 2021 ▸). The starting model was oriented such that the threefold axis was parallel to the *z* axis and passed through the centre of the oligomization domain. Two rigid bodies were then defined for each monomer: residues 3–36 (oligomerization domain) and residues 42–224 (the catalytic domain) taken from PDB entry 7rgv. The position of the oligomerization domain was fixed, and the position and orientation of the catalytic domain were then optimized against the measured scattering data. All 16 models displayed good agreement with the experimental data, but small systematic deviations were apparent. Given these systematic deviations, together with our experience analysing SAXS data for the homologous PmScsC protein (Furlong *et al.*, 2017 ▸), we concluded that the data are consistent with an ensemble of structures in solution. As such, 16 independent ensemble optimizations were performed with *EOM* (Tria *et al.*, 2015 ▸). Each optimization had an initial pool of 1000 structures (all with *C*_3_ symmetry), and the best-fitting ensemble (judged on the basis of the lowest χ^2^) was composed of four structures with diverse conformations, yielding an excellent fit to the data. Thus, the SAXS data are consistent with CcScsC wt being dynamic in solution. Details of the data-collection and structural parameters are summarized in Table 1 ▸, and data have been deposited in the SASBDB (Kikhney *et al.*, 2020 ▸) with accession code SASDLE9.

### 2.10. Crystal structure determination of CcScsC C2S

Reduced CcScsC C2S in SEC buffer was concentrated to 130 mg ml^−1^ and mixed with 2-methyl-2,4-pentanediol (MPD) in the range 37–39%, 0.02 *M* CaCl_2_ and 0.2 *M* sodium acetate pH 7.25 in a 1:1 protein:buffer volumetric ratio in a hanging-drop crystallization setup (1 µl drop equilibrated against 300 µl reservoir solution). Crystallization plates were incubated at 4°C. Crystalline rods grew within five days in these conditions; these were harvested for synchrotron data collection using nylon cryo-loops or litholoops (Molecular Dimensions). Crystals grown in MPD did not require further cryoprotection before flash-cooling in liquid nitrogen.

Diffraction data sets were measured on the MX2 beamline at the Australian Synchrotron equipped with an EIGER 16M detector (Aragão *et al.*, 2018 ▸; supported by the Australian Cancer Research Foundation). The data set that led to the structure reported in this manuscript was collected with a beam attenuation of 30% over a 240° segment corresponding to rotation of the crystal about the φ axis. The data were indexed, integrated and scaled using *XDS* (Kabsch, 2010 ▸). Merging and space-group assignment was achieved with *AIMLESS* and *POINTLESS* (Evans & Murshudov, 2013 ▸) and *Zanuda* (Lebedev & Isupov, 2014 ▸). Molecular replacement was carried out with *Phaser* (McCoy *et al.*, 2007 ▸), using a modified model of PmScsC as the initial search model (PDB entry 4xvw; Furlong *et al.*, 2017 ▸) comprising residues 50–224 of chain *A* with nonconserved residues mutated to alanines. Manual model building in *Coot* (Emsley *et al.*, 2010 ▸) was alternated with automated refinement in *phenix.refine* (Adams *et al.*, 2010 ▸). Due to the high Wilson *B* factor, a sharpening *B* factor of −100 Å^2^ was applied to the maps in the final cycles of building. Reciprocal-space refinement of coordinates and individual *B* factors was carried out using *Phenix*, with weighting optimized for geometric and atomic displacement parameters. Once the *R*_free_ value had decreased below 30%, TLS refinement was also activated using two groups, one consisting of the N-terminal domain (residues 2–63) and the other consisting of the globular domain (residues 64–223). Secondary-structure restraints were enabled for the whole refinement process to ensure tightness of the geometry in α-helical segments placed in regions of poor electron density during model building. The map quality only allowed us to place three water molecules. Evaluation of the quality of the model was performed by *MolProbity* (Chen *et al.*, 2010 ▸). Inspection of the final structure, comparison with other oxidoreductases and generation of figures was carried out using *TM-align* (Zhang & Skolnick, 2005 ▸), *PyMOL* (version 1.8; Schrödinger) and *ChimeraX* (Pettersen *et al.*, 2021 ▸). The side chains of several surface-exposed residues (Lys9, Lys47, Lys97, Arg155, Asp198 and Lys222) were not supported by the electron-density maps and were trimmed to C^β^ or C^γ^ in the model. The coordinates and structure factors have been deposited in the Protein Data Bank (PDB entry 7rgv).

## 3. Results

### 3.1. Mutation of Cys to Ser at position 2 of CcScsC does not affect the *in vitro* isomerase activity

Previously, CcScsC has been shown to rescue at least part of the *E. coli* Δ*dsbC* mutant isomerization defect *in vivo* and was able to refold RNase *in vitro* (Cho *et al.*, 2012 ▸). For our structural studies, we used a variant of CcScsC in which the second residue Cys2 is mutated to Ser (CcScsC C2S). To ensure that the C2S mutation did not affect the enzyme function, we evaluated the isomerase activity of both the wild type and the C2S variant by assessing their ability to refold, and hence reactivate, misfolded RNase. In the assay, each of the enzymes is incubated with scrambled RNase (ScRNase). At specific time points, samples of the enzyme + ScRNase solutions are collected and mixed with cytidine cyclic 2′,3′-monophosphate (cCMP), a substrate of RNase. Active RNase cleaves cCMP, shifting the absorbance intensity, which can be monitored spectrophoto­metrically (a graphical summary of the method is shown in Fig. 2 ▸ *a*).

Our results show that CcScsC activity is unaffected by mutation of Cys2. Both CcScsC wt and CcScsC C2S exhibit an isomerase activity comparable to that of EcDsbC (positive control) in the assay (Fig. 2 ▸ *b*). By comparison, EcDsbA showed a lower and slower isomerase activity in this assay, as expected for a protein classified as an oxidase rather than an isomerase.

### 3.2. CcScsC wt binds copper(I) with subpicomolar affinity

**Figure 3:**
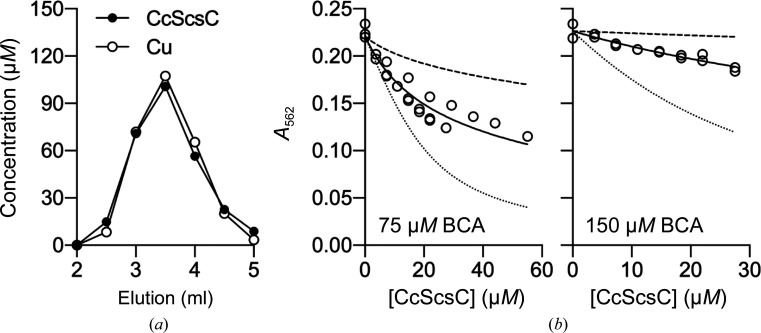
CcScsC wt binds copper(I) with high affinity. (*a*) Co-elution of CcScsC wt with one molar equivalent of copper(I) from a desalting column. The protein concentration in each fraction was determined by an Ellman test. Copper(I) concentrations were determined colorimetrically using bathocuproine disulfonic acid (BCA). (*b*) Titration of CcScsC wt into a mixture of 27–30 µ*M* copper(I) and either 75 µ*M* (*n* = 3) or 150 µ*M* (*n* = 2) BCA. The [Cu^I^BCA_2_]^3−^ complex absorbs light at 562 nm and thus a decrease in *A*_562_ indicates competition between the protein and BCA to bind copper(I). The titration curves were fitted as described in Section 2 to yield log *K*_d_ = 13.1 ± 0.1 *M* (solid lines). Simulated curves corresponding to a tenfold tighter (dotted lines) or tenfold weaker (dashed lines) affinity are displayed as references.

*S.* Typhimurium ScsB and ScsC proteins have both been reported to bind copper(I) with subpicomolar affinity, modulating the copper tolerance through the sequestration of excess copper and interaction with other copper-binding proteins such as CueP, for example (Subedi *et al.*, 2019 ▸). Here, we investigated the ability of CcScsC wt to bind copper. Based on co-elution of Cu with CcScsC wt on a desalting column, we determined that reduced CcScsC wt binds copper(I) with a 1:1 protein:copper(I) stoichiometry (Fig. 3 ▸ *a*). By competing this protein against the colorimetric copper(I) ligand 2,2′-bicinchoninic acid (BCA), we found that reduced CcScsC wt exhibits a subpicomolar affinity for copper(I), with a log *K*_d_ of −13.1 ± 0.1 *M* (Fig. 3 ▸ *b*).

### 3.3. CcScsC restores the swarming-motility defect in a *P. mirabilis* mutant lacking the native trimeric isomerase PmScsC

**Figure 4:**
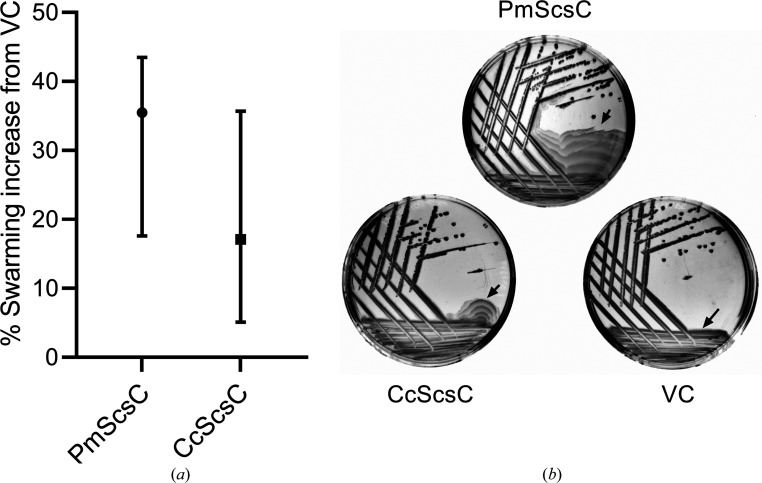
CcScsC wt functionally complements PmScsC in *P. mirabilis* swarming-motility assays. *P. mirabilis* strain PM54 with *scsC* deleted (PM54Δ*scsC*; Furlong *et al.*, 2017 ▸) was complemented with pSU2718 plasmid vectors expressing PmScsC, CcScsC wt or the empty vector control (VC). Strains were assessed for swarming motility on the surface of LB agar in the presence of copper (*a*) or in the absence of sodium chloride (*b*), as detailed in Section 2 and described previously (Subedi *et al.*, 2021 ▸; Furlong *et al.*, 2017 ▸). (*a*) The swarming surface area was measured for each strain after 24 h of incubation at 37°C on LB Lennox agar plates containing 1.6 m*M* CuSO_4_, 100 µ*M* IPTG and 17 µg ml^−1^ chloramphenicol and expressed as the percentage swarming increase over PM54Δ*scsC* vector control (VC). The median with interquartile range for the percentage swarming increase over the VC is plotted for each strain from eight biological repeats. (*b*) Swarming-plate images of each strain streaked on NaCl-free LB agar. Plates were incubated at 37°C for 24 h in NaCl-free LB agar (1.5% agar) supplemented with 100 µ*M* IPTG and 17 µg ml^−1^ chloramphenicol. The swarming-motility fronts are marked by black arrows. Images are representative of three independent biological repeats.

Our previous work demonstrated that PmScsC is required for *P. mirabilis* swarming motility in the presence of copper(II) (Furlong *et al.*, 2017 ▸). The similarity in the sequences of CcScsC and PmScsC (Fig. 1 ▸) and the isomerase activity of CcScsC (Fig. 2 ▸) prompted us to test whether these similarities extended to *in vivo* function. For this, we complemented a previously characterized *P. mirabilis* Δ*scsC* mutant with CcScsC wt or with native PmScsC and assessed bacterial swarming motility on LB media containing copper(II) (Furlong *et al.*, 2017 ▸). Complementation with PmScsC enabled swarming of the Δ*scsC* mutant, as reported previously (Fig. 4 ▸ *a*). Complementation with CcScsC wt also restored swarming in the Δ*scsC* mutant under these conditions (Fig. 4 ▸ *a*). As swarming can be sensitive to assay conditions (indicated by the relatively large data variability reported in Fig. 4 ▸ *a*), we also tested for functional complementation in an assay that monitors *P. mirabilis* Δ*scsC* swarming recovery on the surface of LB agar lacking NaCl. The sodium chloride requirement for swarming was previously shown to depend on PmScsC in *P. mirabilis* and another trimeric protein StBcfH in *S.* Typhimurium (Subedi *et al.*, 2021 ▸). Similar to swarming in the presence of copper, both PmScsC and CcScsC wt restored the swarming defect in Δ*scsC* carrying an empty vector control (VC; Fig. 4 ▸ *b*). Collectively, the shared ability of CcScsC wt and PmScsC to restore swarming in *P. mirabilis* under two independent assay conditions suggests that they can mediate the folding of the same substrate(s) *in vivo*.

### 3.4. Mass photometry shows that CcScsC is trimeric in solution

The shared ability of CcScsC wt and PmScsC to restore swarming in *P. mirabilis* may in part be facilitated by a shared architecture, and therefore we next sought to determine whether CcScsC is dimeric (as reported previously on the basis of SEC analysis; Cho *et al.*, 2012 ▸) or trimeric like PmScsC (Furlong *et al.*, 2017 ▸). Mass photometry measures the distribution of the molecular masses of proteins and other large particles in solution in their native state without the need to label proteins (Young *et al.*, 2018 ▸). As proteins in solution bind to a thin glass slide, they cause laser light to scatter with an intensity proportional to their molecular mass: the heavier the protein, the larger the scattering signal. Single binding events are recorded over a predefined period of time to generate a histogram that reflects the mass distribution of the particles in the sample. Mass photometry can therefore be used to determine the molecular mass of single proteins (with a molecular weight greater than 40 kDa), protein complexes and oligomers (Wu & Piszczek, 2021 ▸). Using this technique, the oligomeric state of CcScsC was determined without the need for labelling.

**Figure 5:**
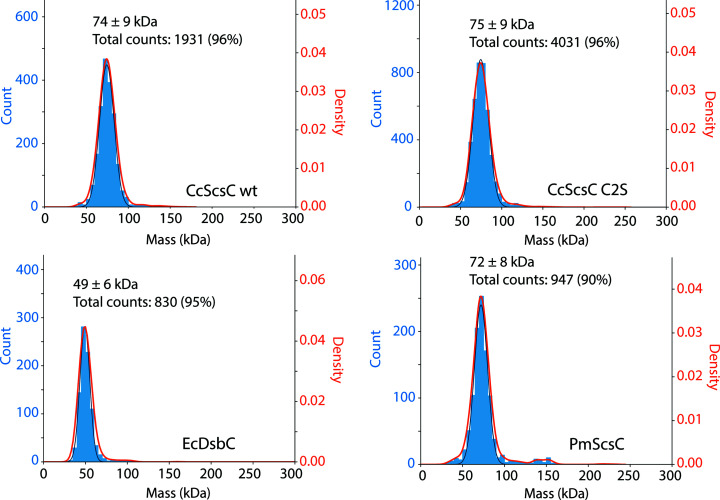
Mass-photometry experiments indicate that CcScsC forms a trimer in solution. Each panel represents the mass distribution for the indicated sample (CcScsC wt and CcScsC C2S); EcDsbC and PmScsC were used as dimeric and trimeric thioredoxin-fold protein controls. Proteins were diluted to 150 n*M* and slowly added to reference buffer (25 m*M* HEPES pH 7.5, 150 m*M* NaCl) until around 1000 binding events per minute were observed and recorded. The mean mass of each peak, the standard deviation and the total number of events included in the peak (percentage of total events) are reported at the top of each peak. The peak positions had average masses of 75 ± 9 and 74 ± 9 kDa for CcScsC C2S and CcScsC wt, respectively, indicating a trimeric state in solution (the mass of a monomer is 25.1 kDa). Controls were included as a reference: EcDsbC showed a peak at 49 kDa corresponding to a dimer (24.1 kDa monomer) and PmScsC showed a peak at 72 kDa corresponding to a trimer (24.5 kDa monomer) consistent with previous crystal structure determinations (Furlong *et al.*, 2017 ▸; Zapun *et al.*, 1995 ▸).

Samples of the reduced proteins CcScsC wt and CcScsC C2S were evaluated by mass photometry. CcScsC wt produced a single peak with scattering corresponding to a mean molecular mass of 75 kDa with a standard deviation of ±9 kDa. Similar results were generated for CcScsC C2S, with a mean molecular mass of 74 ± 9 kDa. The expected molecular weight for a trimer is 75.3 kDa (the mass of a monomer is 25.1 kDa and a dimer would be 50.2 kDa; Fig. 5 ▸). The results were robust (over 4000 events and 2000 events recorded for CcScsC C2S and CcScsC wt, respectively), indicating that the protein is trimeric in solution. Control proteins were also evaluated: EcDsbC, a known protein dimer, revealed a peak at 49 ± 6 kDa (24.1 kDa monomer; Zapun *et al.*, 1995 ▸) and trimeric PmScsC displayed a peak at 72 ± 8 kDa (24.5 kDa monomer; Furlong *et al.*, 2017 ▸), demonstrating that the technique correctly identified the known oligomerization states of these two thioredoxin-fold proteins that have previously been characterized by protein crystallography.

### 3.5. Crystal structure of CcScsC C2S

We crystallized purified CcScsC C2S protein at 4°C from a solution consisting of 39% 2-methyl-2,4-pentanediol (MPD), 20 m*M* CaCl_2_, 200 m*M* sodium acetate buffer pH 7.25. The protein formed long hexagonal crystal rods over the course of a week. Crystals were sent to the Australian Synchrotron for data collection. The X-ray diffraction data measured from a single crystal were indexed and the data were integrated in space group *P*6_3_, with unit-cell parameters *a* = *b* = 114.0, *c* = 48.7 Å, α = β = 90, γ = 120°. The diffraction data showed some deviation from the Wilson plot (around 13% of all bins deviated from the theoretical values) and the experimental Wilson *B* factor was very high: close to 90 Å^2^. No twinning or translational noncrystallographic symmetry was detected.

The structure was solved by molecular replacement using the globular domain of the compact structure of PmScsC as a search model. While molecular replacement yielded a solution relatively easily (comprising one protomer per asymmetric unit), rebuilding the model proved challenging. The *R*_free_ values of the initial models remained stubbornly high (>45%). Attempts to solve the structure in a different space group did not improve the outcome. The unit cell had a high solvent content (∼65%) and the electron-density maps were of moderate quality (possibly due to the sparsity of interdomain and interchain contacts). We built in side chains starting from the most conserved and well defined regions (the C*XX*C active site and *cis*-proline), and used secondary-structure constraints and map sharpening and avoided real-space refinement. The final model was refined to an *R*_free_ value of 0.251 (Table 2 ▸).

**Figure 6:**
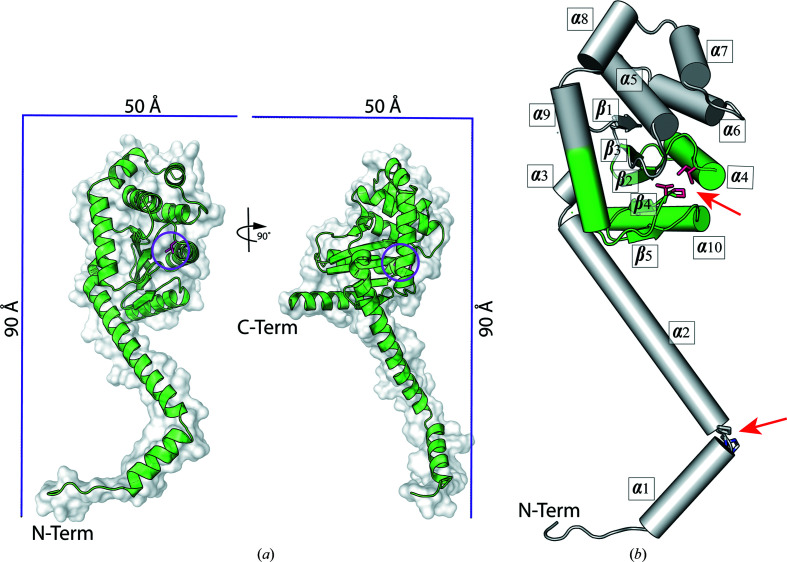
Crystal structure of the reduced CcScsC C2S protomer. (*a*) Model of the CcScsC C2S protomer in a combination of cartoon and surface representations. CcScsC C2S is composed of a long N-terminal α-helix (N-term) and a globular domain. The structure is rotated 90° along its long axis to facilitate visualization of the whole protein, including its C-terminal α-helix (C-term). The scale box provides a reference for the size of the protein. The active site is highlighted by a pink circle. (*b*) Secondary-structure elements of CcScsC C2S. The protein is comprised of ten α-helices: three in the N-terminal tail and seven in the globular domain. Residues His22 and Pro23, which are part of the bend in the N-terminal domain, are shown as sticks and highlighted with a red arrow between helices α1 and α2. The globular domain supports a typical thioredoxin fold, coloured green, with the two catalytic cysteines represented as pink sticks facing Thr191–*cis*-Pro192, which is also shown in pink and highlighted with a red arrow.

**Figure 7:**
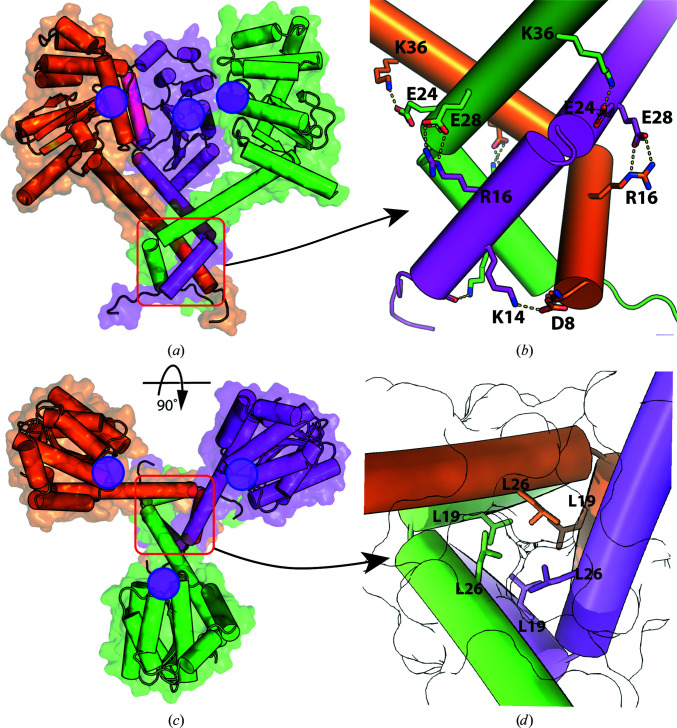
Trimerization of CcScsC. (*a*) Side view of the crystallographic trimer with the three protomer chains represented as surfaces and cartoons coloured orange, magenta and green. The trimer is approximately 80 Å in width. It is held together via interaction of the N-terminal α-helices (red box). (*b*) Close-up of the trimerization domain viewed from the side [red box in (*a*)] including electrostatic interactions between Asp8 and Lys14, Arg16 and Glu28, and Glu24 and Lys36. (*c*) Top view of the crystallographic trimer and (*d*) close-up of the inside of the trimerization domain [red box in (*c*)]. The interior of the trimer is lined with hydrophobic residues, including leucines (Leu19 and Leu26 are shown as sticks). The surface of each protomer is outlined in black, demonstrating the tight packing of the residues inside the helices. The positions of the active sites on the CcScsC C2S protomers are indicated with filled magenta circles. Models are shown as a combination of cartoon and surface representations and important residues are shown as sticks and labelled in (*b*) and (*d*). Electrostatic interactions are shown as dashed lines.

The model of the crystal structure of the reduced CcScsC C2S protomer is presented in Fig. 6 ▸. The protein has an extended N-terminal α-helix with a bend in the helix between His22 and Pro23 (Fig. 6 ▸ *b*). The α-helix joins the globular domain of the protein at Tyr52. The globular domain of the CcScsC C2S protomer is a typical thioredoxin fold with two catalytic cysteines (positions 81 and 84; pink sticks in Fig. 6 ▸) in close proximity to a *cis*-Pro motif (Thr191–*cis*-Pro192). Overall, the protomer forms ten α-helices and five β-strands arranged similarly to those in the crystal structure of the extended form of PmScsC (PDB entry 5id4; Furlong *et al.*, 2017 ▸). In the unit cell, six protomers are organized by crystallographic symmetry into two trimers. Each trimer is formed from the interaction of the N-terminal α-helical regions of three protomers (Fig. 7 ▸). Hydrophobic contacts line the interior of the trimer interface (Fig. 7 ▸ *d*). On the external surface of the trimer, the side chains of several pairs of N-terminal residues located on neighbouring protomers are within electrostatic interaction distance (Asp8 and Lys14, Asp16 and Glu28, and Glu24 and Lys36; Fig. 7 ▸). An equivalent to the Arg16–Glu28 interaction has also been identified in the crystal structure of PmScsC (Arg18–Glu30; Furlong *et al.*, 2019 ▸).

**Figure 8:**
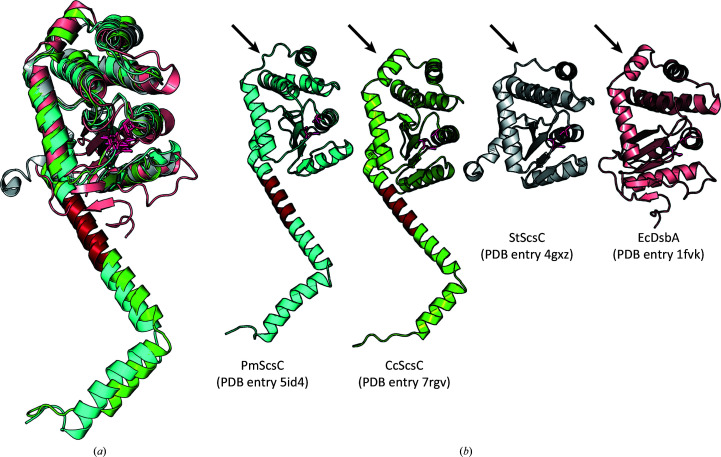
Comparison of the protomers of CcScsC C2S, PmScsC (extended), StScsC and EcDsbA. (*a*) Superimposition of the globular domains of the four oxidoreductases shows the structural conservation between these proteins: EcDsbA (PDB entry 1fvk; Guddat *et al.*, 1997 ▸) in light pink, StScsC (PDB entry 4gxz; Shepherd *et al.*, 2013 ▸) in grey, PmScsC (PDB entry 5id4; Furlong *et al.*, 2017 ▸) in cyan and CcScsC (PDB entry 7rgv) in green. In each case, the C*XX*C and *cis*-proline loop residues are shown as pink sticks. (*b*) The same structures are displayed side by side showing the α-helix/loop difference in the globular domains (arrow). The sequence identified to be responsible for the flexible, dynamic nature of PmScsC is highlighted in red. The homologous region of CcScsC is also coloured red.

There are three reported crystal structures of the PmScsC protomer and trimer: compact, intermediate and extended (Furlong *et al.*, 2017 ▸). The crystal structure of the CcScsC C2S protomer closely resembles that of the extended structure of PmScsC (PDB entry 5id4; Furlong *et al.*, 2017 ▸; Fig. 8 ▸). The two protein structures align with an r.m.s.d. of 2.41 Å (214 C^α^ atoms aligned with *TM-align*; Zhang & Skolnick, 2005 ▸). The main differences are the presence of the α8 helix (globular domain; arrow in Fig. 8 ▸ *b*), which is absent in PmScsC but often present in DsbA structures. The importance of this helix is unclear.

The structure of the globular domain of the CcScsC C2S protomer aligns well with that of StScsC (r.m.s.d. of 1.94 Å for 168 C^α^ atoms aligned with *TM-align*; Zhang & Skolnick, 2005 ▸). The largest difference is the presence of the N-terminal helical domain and helix α8 in CcScsC (Fig. 8 ▸ *b*), which are absent in StScsC. The globular domain of the CcScsC C2S protomer aligns less well with EcDsbA (r.m.s.d. of 3.34 Å for 163 C^α^ atoms aligned with *TM-align*; Zhang & Skolnick, 2005 ▸) although both have a helix at the α8 position.

PmScsC is a dynamic protein that is able to adopt multiple conformations in solution that are captured in compact, extended and intermediate structures in the crystal (Furlong *et al.*, 2017 ▸). The dynamic nature of the protein is dependent on the presence of a short 12-residue stretch of sequence that is rich in Lys (2), Gln (4) and Ala (2) residues (residues ^38^KKADEQQAQFRQ^49^) linking the N-terminal helix to the globular catalytic domain (Furlong *et al.*, 2019 ▸). This linker adopts different secondary structures in solution, disordered, β-strands and α-helical, dependent on noncovalent inter­actions with nearby residues in the globular domain and in other protomers (Smith *et al.*, 2021 ▸). The N-terminal region of the CcScsC C2S protomer has a similar sequence enriched in Lys (2), Gln (4) and Ala (3) residues (^36^KQAAQQAVSSQK^47^; the region highlighted in red in Fig. 8 ▸ *b*) preceding the globular domain. The similarity between the sequences suggests that CcScsC might have a comparable dynamic quality to PmScsC.

### 3.6. Small-angle X-ray scattering data are consistent with a dynamic CcScsC trimer

**Figure 9:**
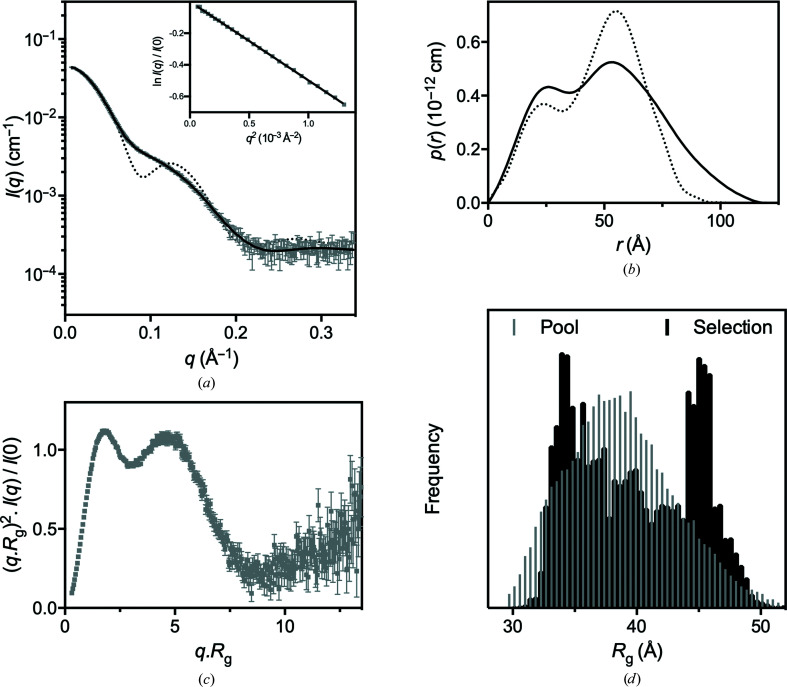
Small-angle X-ray scattering data. (*a*) SAXS data collected from CcScsC wt (grey) with the calculated scattering profile of the ensemble model overlaid in black (SASBDB entry SASDLE9). The predicted scattering profile of the crystal structure is also shown (dotted line; PDB entry 7rgv). The agreement between the experimental data and the ensemble model is excellent, yielding χ^2^ = 1.32 (*CorMap* test *P* = 0.917) relative to the static rigid-body model (χ^2^ = 1.95; *CorMap* test *P* = 0.000) and the calculated scattering profile from the crystal structure (χ^2^ = 164; *CorMap* test *P* = 0.000). The Guinier region (inset) of the scattering data is linear, consistent with a monodisperse solution. (*b*) Pair-distance distribution function [*p*(*r*)] derived from the scattering data (solid line), showing that the maximum dimension of the particles in solution is 120 Å compared with 80 Å in the crystal trimer. Also shown is the calculated *p*(*r*) for the crystal structure (dotted line), which differs significantly from the experimentally determined SAXS solution *p*(*r*) curve. (*c*) The dimensionless Kratky plot calculated for the SAXS data. The first peak is consistent with a largely globular protein complex, while the second peak is due to the globular catalytic domains. At larger values of *qR*_g_ the plot tends upwards, which is indicative of a flexible protein. (*d*) A histogram of the frequency as a function of the radius of gyration of the pool of structures (grey) and the selected structures (black). This plot shows that the ensemble analysis has preferentially selected structures with *R*_g_ values of ∼35 and ∼45 Å relative to the pool population. This bimodal distribution is indicative of a molecule that is present mostly as a compact or extended molecule in solution.

To assess the low-resolution solution structure of CcScsC, small-angle X-ray scattering was measured from a dilute solution of CcScsC wt (Table 1 ▸). The measurements were made on the SAXS/WAXS beamline at the Australian Synchrotron with an inline SEC setup which separates aggregate and other impurities from the protein of interest immediately prior to measurement. The resulting data are of excellent quality (as judged by the high signal to noise and the linear Guinier region; Fig. 9 ▸ *a*, inset). The Porod analysis is consistent with the protein being trimeric in solution, in line with the mass-photometry results and the crystal structure. However, both the radius of gyration (*R*_g_) and the maximum particle dimension (*D*_max_) determined from the scattering data are significantly larger than those predicted from the crystal structure (Table 1 ▸). This difference is further highlighted by a comparison of the experimental scattering data and pair-distance distribution function obtained from the experimental data with those derived from the crystal structure (dotted lines in Figs. 9 ▸ *a* and 9 ▸ *b*). The structure of the trimer in solution is therefore shown to be less compact than the trimer in the crystal structure. This difference could result from the presence of a single structure that differs from the crystal structure or from the presence of numerous conformations in solution, as observed previously for PmScsC (Furlong *et al.*, 2017 ▸).

Two modelling approaches were taken to further interpret the SAXS data. The first approach was to take the trimerization and catalytic domains from the CcScsC C2S crystal structure to optimize a single rigid-body model against the scattering data. The best resulting model was a good fit to the experimental data, but there were clear systematic deviations between the model and the data. The second approach was to use an ensemble-modelling approach, as was employed to model the PmScsC SAXS data (Furlong *et al.*, 2017 ▸). For the CcScsC wt data, an ensemble of four diverse structures was sufficient to provide an excellent fit to the scattering data (Fig. 9 ▸ *a*). While we captured a single conformation of CcScsC C2S in the crystal structure, the comparison between the scattering profiles predicted from the crystal structure and the experimental SAXS data provides strong evidence that the protein trimer can sample multiple conformational states in solution. Further, the upturn in the Kratky plot (Fig. 9 ▸ *c*) at large values of *qR*_g_ and the bimodal nature of the *R*_g_ selection pool frequency from the ensemble analysis (Fig. 9 ▸ *d*) are both consistent with a dynamic protein. Taken together, the SAXS data analysis and modelling suggest that CcScsC, like PmScsC, is dynamic in solution.

## 4. Discussion

SCS proteins, in particular ScsB and ScsC, appear to be involved in copper-resistance pathways in bacteria. Moreover, the ScsB and ScsC proteins share similarities with the DSB proteins DsbD and DsbC, respectively. Because they are essential for the folding of several virulence factors in the periplasm of bacteria, DSB proteins have been investigated as potential targets for drug discovery (Landeta *et al.*, 2017 ▸; Bocian-Ostrzycka *et al.*, 2017 ▸; Halili *et al.*, 2015 ▸; Duprez *et al.*, 2015 ▸; Vezina *et al.*, 2020 ▸; Nebl *et al.*, 2020 ▸; Furniss *et al.*, 2022 ▸). Expanding our understanding of the related SCS proteins, including their mode of action and structural variation, may contribute to the identification of new druggable targets to fight bacterial infections.

The functional and structural characterization experiments described here were performed using the wild-type CcScsC protein, with the exception of the crystallization studies, in which only the C2S variant formed crystals suitable for X-ray analysis. One possible explanation for this is that CcScsC wt may form a disulfide bond between the N-terminal cysteines at the high concentration required for crystallization (above 100 mg ml^−1^, well above physiological levels) and this may interfere with the crystallization process. Replacing Cys2 with Ser in the C2S variant does not affect the disulfide isomerase activity (Fig. 2 ▸) or trimerization (Fig. 5 ▸) of the protein.

Overall, we found that the crystal structure of CcScsC C2S closely resembles the extended conformation of PmScsC (PDB entry 5id4; Furlong *et al.*, 2017 ▸). The long N-terminal trimerization domain oligomerizes three protomers via hydrophobic interactions of residues lining the interior of the N-terminal α-helix and surface electrostatic inter­actions, most notably the Arg16–Glu28 interaction that is conserved in PmScsC.

We confirmed that CcScsC wt and its C2S mutant are efficient isomerases, with an activity comparable to that of EcDsbC in refolding scrambled bovine RNase. We also demonstrated that CcScsC wt binds copper with a sub­picomolar affinity similar to that observed for StScsC (Subedi *et al.*, 2019 ▸). *C. crescentus* possesses the Pco system for the detoxification of periplasmic copper (Lawarée *et al.*, 2016 ▸). While the *C. crescentus* proteins have not been characterized biochemically, the PcoC family of proteins are known to bind copper(I) with a *K*_d_ of ∼10^−13^ *M* (Xiao & Wedd, 2010 ▸), similar to the affinity that we measured for CcScsC. Thus, CcScsC may help to counter copper stress in the periplasm using two different mechanisms: (i) through its isomerase activity by repairing the damage caused by nonspecific oxidation of cysteine-containing proteins and (ii) by sequestering copper(I) and supporting the action of the Pco periplasmic copper-detoxification system. A role for CcScsC in countering copper stress is supported by our *in vivo* studies, in which CcScsC rescues *P. mirabilis* Δ*scsC* swarming under copper stress.

It is now clear that bacterial disulfide oxidoreductases can be monomeric [for example EcDsbA (Martin *et al.*, 1993 ▸) and StScsC (Shepherd *et al.*, 2013 ▸)], dimeric [for example DsbC (McCarthy *et al.*, 2000 ▸) and DsbG (Heras *et al.*, 2004 ▸)] or trimeric [for example CcScsC, PmScsC (Furlong *et al.*, 2017 ▸), WpDsbA2 (Walden *et al.*, 2019 ▸) and StBcfH (Subedi *et al.*, 2021 ▸)]. In EcDsbC the dimerization domain contributes to the selectivity of protein partners: removal of the N-terminal dimerization domain of EcDsbC resulted in a protein with oxidase activity that cross-reacts with *E. coli* DsbB (the redox partner of EcDsbA; Bader *et al.*, 2001 ▸). The function of the oligomerization domain of trimeric thioredoxin-fold proteins is less clear. In PmScsC, the N-terminal helix has a short segment that provides flexibility. It has been suggested that this segment allows the three catalytic domains of PmScsC to explore a wide range of conformations during the refolding of bound misfolded substrates (Furlong *et al.*, 2017 ▸, 2019 ▸). Trimeric StBcfH adopts at least two different conformations (found in the crystal asymmetric unit), and like eukaryotic protein disulfide isomerases (Soares Moretti & Martins Laurindo, 2017 ▸) StBcfH is both a dithiol oxidase and a disulfide isomerase (Subedi *et al.*, 2021 ▸). For trimeric WpDsbA2, SAXS data analysis suggests that the N-terminal trimerization domain is rigid and may contribute to disulfide isomerase activity simply by bringing three DsbA-like domains into close proximity (Walden *et al.*, 2019 ▸). For the three Scs proteins, StScsC [monomeric, not an oxidase or an isomerase, binds copper(I)], PmScsC (trimeric, an isomerase) and CcScsC [trimeric, an isomerase, binds copper(I)] have structurally similar catalytic domains: StScsC lacks the N-terminal domain that enables trimerization of the PmScsC and CcScsC proteins, and this is likely to contribute to its different function.

In conclusion, we have demonstrated that CcScsC adopts a trimeric conformation in solution and in the crystal structure. The trimer, which is formed by the interaction of the N-terminal regions of three protomers, is dynamic in solution. The structural similarities between PmScsC and CcScsC are reflected by *in vitro* and *in vivo* functional similarities: they are both protein disulfide isomerases and they both support swarming in *P. mirabilis* under two independent assay conditions. Our functional and structural characterization of CcScsC expands our understanding of the structurally related DSB disulfide-bond and SCS copper-resistance systems and how these intertwined systems help bacteria thrive in stressful environments.

## Supplementary Material

PDB reference: suppressor of copper sensitivity protein C, 7rgv

SASBDB reference: suppressor of copper sensitivity protein C, SASDLE9

## Figures and Tables

**Table 1:** SAXS data-collection and analysis details for CcScsC wt

Data-collection parameters	
Instrument	SAXS/WAXS (Australian Synchrotron)
Beam geometry (µm)	80 (vertical) × 200 (horizontal)
Wavelength (Å)	1.078
Camera length (m)	2.791
*q*-range (Å^−1^)	0.006–0.50
Exposure time (s)	28 (28 × 1 s exposures)†
Configuration	SEC-SAXS (S200 5/150 GL) with sheath flow
Injection concentration (mg ml^−1^)	5.6
Injection volume (µl)	60
Flow rate (ml min^−1^)	0.45
Temperature (K)	283
Absolute intensity calibration	Water
Sample details	
Extinction coefficient (*A*_280_, 0.1%)	0.252
Partial specific volume (cm^3^ g^−1^)	0.739
Contrast Δρ (10^10^ cm^−2^)	2.878
Molecular mass (kDa)	75 (trimer)
Average protein concentration (mg ml^−1^)	∼1.7‡
Structural parameters	
*I*(0) (cm^−1^) (from Guinier)	0.04517 ± 0.00008
*R*_g_ (Å) (from Guinier)	38.7 ± 0.1
*I*(0) (cm^−1^) [from *p*(*r*)]	0.04534 ± 0.00012
*R*_g_ (Å) [from *p*(*r*)]	39.0 ± 0.1
*D*_max_ (Å)	120 ± 6
Porod volume (Å^3^)	96500 ± 5000
*R*_g_ (Å) (crystal structure)	35.7
*D*_max_ (Å) (crystal structure)	103
Dry volume (Å^3^) (from sequence)	90000
Molecular-mass determination	
Molecular mass (kDa) (from Porod)	79 ± 4
Software employed	
Primary data reduction	*scatterBrain* (version 2.71)
Data processing	*PRIMUS* (version 3.2) and *GNOM* (version 4.6)
Scattering-profile calculation	*CRYSOL* (version 2.8.3)
Rigid-body modelling	*CORAL* (version 1.1) and *EOM* (version 2.0)

† The *R*_g_ across the 28 averaged frames shows no systematic trend; hence, it is deemed that there are no significant interparticle interactions present in the data.

‡ The actual protein concentration was not measured; the value shown is calculated from *I*(0), assuming a molecular mass of 75 kDa.

**Table 2:** Data-collection and refinement statistics for the model of CcScsC (PDB entry 7rgv) Values in parentheses are for the highest resolution shell.

Wavelength (Å)	0.9536
Resolution range (Å)	37.0–2.63 (2.74–2.63)
Space group	*P*6_3_
*a*, *b*, *c* (Å)	114.0, 114.0, 48.7
α, β, γ (°)	90, 90, 120
Molecules per asymmetric unit	1
Total reflections	130647 (12884)
Unique reflections	10854 (1045)
Multiplicity	12.0 (12.3)
Completeness (%)	98.75 (94.76)
Mean *I*/σ(*I*)	22.51 (1.85)
Wilson *B* factor (Å^2^)	90
*R*_merge_	0.052 (1.46)
*R*_meas_	0.054 (1.52)
*R*_p.i.m._	0.016 (0.431)
CC_1/2_	1 (0.961)
CC*†	1 (0.99)
Reflections used in refinement	10767 (1030)
Reflections used for *R*_free_	1083 (99)
*R*_work_	0.223 (0.404)
*R*_free_	0.251 (0.430)
CC_work_	0.975 (0.841)
CC_free_	0.941 (0.756)
No. of non-H atoms	
Total	1676
Protein	1673
Water	3
Protein residues	222
R.m.s.d., bond lengths (Å)	0.002
R.m.s.d., angles (°)	0.45
Ramachandran favoured (%)	96.8
Ramachandran allowed (%)	3.2
Ramachandran outliers (%)	0.00
Rotamer outliers (%)	1.2
Clashscore	2.4
Average *B* factor (Å^2^)	
Overall	122
Protein	122
Water	116
No. of TLS groups	2

† CC* = [2CC_1/2_/(1 + CC_1/2_)]^1/2^ (Karplus & Diederichs, 2012 ▸).
